# Combating Single-Frequency Jamming through a Multi-Frequency, Multi-Constellation Software Receiver: A Case Study for Maritime Navigation in the Gulf of Finland

**DOI:** 10.3390/s22062294

**Published:** 2022-03-16

**Authors:** Saiful Islam, Mohammad Zahidul H. Bhuiyan, Sarang Thombre, Sanna Kaasalainen

**Affiliations:** 1 Finnish Geospatial Research Institute (FGI-NLS), 02430 Kirkkonummi, Finland; zahidul.bhuiyan@nls.fi (M.Z.H.B.); sarang.thombre@nls.fi (S.T.); sanna.kaasalainen@nls.fi (S.K.); 2 u-blox Espoo Oy, 02600 Espoo, Finland

**Keywords:** GNSS vulnerability, jamming, satellite navigation

## Abstract

Today, a substantial portion of global trade is carried by sea. Consequently, the reliance on Global Navigation Satellite System (GNSS)-based navigation in the oceans and inland waterways has been rapidly growing. GNSS is vulnerable to various radio frequency interference. The objective of this research is to propose a resilient Multi-Frequency, Multi-Constellation (MFMC) receiver in the context of maritime navigation to identify any GNSS signal jamming incident and switch to a jamming-free signal immediately. With that goal in mind, the authors implemented a jamming event detector that can identify the start, end, and total duration of the detected jamming event on any of the impacted GNSS signal(s). By utilizing a jamming event detector, the proposed resilient MFMC receiver indeed provides a seamless positioning solution in the event of single-frequency jamming on either the lower or upper L-band. In addition, this manuscript also contains positioning performance analysis of GPS-L5-only, Galileo-E5a-only, and Galileo-E5b-only signals and their multi-GNSS combinations in a maritime operational environment in the Gulf of Finland. The positioning performance of lower L-band GNSS signals in a maritime environment has not been thoroughly investigated as per the authors’ knowledge.

## 1. Introduction

Positioning, Navigation, and Timing (PNT) are critical components of modern technology systems that we rely on every day. Global Navigation Satellite Systems (GNSSs) revolutionize the world by delivering precise PNT in good signal conditions. In the consumer segment, the maritime industry was one of the first communities to adopt satellite-based navigation. GNSS have become the primary source of PNT for the integrated Electronic Chart Display and Information Systems (ECDIS) that are broadly used on commercial and recreational vessels [1]. GNSS is also used to ensure safe navigation in inland waterways, coastal waters, port approaches, harbor entrances, as well as search and rescue operations in the ocean [2]. Further, GNSS is used to assist with Under-Keel Clearance (UKC) management using Real-Time Kinematic (RTK) receivers [3]. However, due to low signal strength and the lack of authentication on legacy signals, GNSS is vulnerable to various radio frequency interference (i.e., jamming/spoofing). With the rising availability of low-cost jamming devices in the last decade, there has been a lot of concern on the reliable use of GNSS signals. The maritime industry is among the first to be affected by such deliberate disruptions. Hundreds of GNSS jamming events were reported last year in seashore environments, particularly in the Mediterranean Sea, Black Sea, and Suez Canal, etc. [4,5]. The U.S Coast Guard Navigation Center’s (NAVCEN) website [6] has a historical record of GPS interruptions reported by maritime users. Recent incidents serve as a reminder that availability, reliability, and continuity are critical, and even a slight degradation in signal quality may result in safety hazards and outages that can cost invaluably.

Currently, there are four global GNSS systems that transmit PNT signals in multiple-frequency bands. Therefore, modern GNSS receivers could take advantage of Multi-Frequency, Multi-Constellation (MFMC) diversity to offer a reliable PNT solution in the event of signal disruption in one of the frequency bands. The MFMC diversity will increase the availability, reliability, and continuity of PNT solution if utilized intelligently.

In [7], author presented an analysis of multi-GNSS Precise Point Positioning (PPP) performance in the maritime environment. The number of satellites per constellation, Dilution of Precision (DOP), and 2-Dimentional positioning accuracy were analyzed using a professional-grade maritime receiver. The article did not assess the impact of jamming on positioning accuracy. In [8], researchers demonstrated single- and multi-GNSS performance in terms of satellite coverage and availability using GNSS data from International GNSS Service (IGS) stations in coastal areas.

Articles [9,10,11] investigated the detection of radio frequency interference and related threats in the maritime environment. The studies were mostly focused on the detection and mitigation of intentional and unintentional interference. The impact and consequence of real-life jamming on GPS signals in the maritime environment were discussed in [12]. The authors proposed an Enhanced Loran (eLoran)-based system diversification to reduce GNSS vulnerability in case of signal disruption. The impact of jamming on maritime GNSS receivers in Norwegian shore was addressed in [13]. The study also presented partial positioning performance assessments of different GNSS signals, particularly GPS L1 and GLONASS G1. So far, there have been limited results presented in the literature about the impact of jamming on the positioning accuracy of a marine vessel in a real-life operational environment.

The objective of this research is to immediately identify any jamming incident on any of the GNSS signals and to propose a resilient MFMC receiver to offer an uninterrupted positioning solution in the event of single-frequency or single-constellation jamming. The authors first implement a jamming event detector based on received signal strength along with other modifications of a GNSS software receiver. Furthermore, the influence of jamming on legacy GNSS signals received on the L1/E1/B1 frequency bands is presented. The impact of jamming on positioning accuracy is also analyzed separately on L5/E5 frequency band. This manuscript then presents a comprehensive positioning performance assessment of individual GNSS constellation for four different scenarios: (i) nominal maritime navigation without the presence of jamming, (ii) maritime navigation under the influence of jamming on GNSS L1/E1/B1 signal, (iii) maritime navigation under the influence of jamming on GNSS L5/E5a/E5b signal, (iv) maritime navigation under the influence of jamming on lower and upper L-bands. Finally, the authors proposed a resilient MFMC receiver based on the implemented jamming event detector. The findings imply that, in the event of single-frequency jamming, the resilient MFMC receiver can work seamlessly by switching to the jamming-free GNSS signals using a jamming event detector. In the case of multi-frequency jamming, the proposed resilient MFMC receiver falls back to a traditional receiver with whatever GNSS signals it can track and tries to offer position solutions with those measurements.

The jamming test campaign was carried out on a cruise ship named “MS Megastar” from Tallink in the Gulf of Finland, which is the easternmost arm of the Baltic Sea. An in-house software receiver known as “FGI-GSRx” [14,15,16,17] is used to process and analyze the raw samples recorded by the radio front-end. In addition to FGI-GSRx, separate MATLAB scripts are utilized to analyze the results and generate appropriate figures. The other novelty of this work is the analysis of positioning performance for GPS-L5-only, Galileo-E5a-only, or Galileo-E5b-only signals in a maritime operational environment. To the authors’ knowledge, there is little analysis present in the literature for comparing the performance of each signal on the L5/E5 band along with the legacy L-band signals (i.e., L1/E1/B1) for maritime navigation.

The remainder of this manuscript is organized as follows: Section 2 describes the processing software, definition for jamming event detection, hardware setup for data collection, and the associated front-end configuration. Section 3 provides an overview and concept illustration of the proposed resilient MFMC GNSS receiver. Section 4 demonstrates four different test scenarios that were stated earlier in this section. Section 5 discusses the performance of several GNSS signals in four different test scenarios. Finally, Section 6 concludes the manuscript with the findings of the experimental analysis and some guidelines for future work.

## 2. Materials and Methods

### 2.1. FGI-GSRx Multi-Frequency, Multi-Constellation Receiver

FGI-GSRx is a MATLAB-based Software-Defined Receiver (SDR) developed at the Finnish Geospatial Research Institute (FGI). It is a MFMC software receiver used to test and validate novel receiver processing algorithms for resilient and accurate GNSS positioning performance. At present, FGI-GSRx can process GNSS signals from multiple constellations, including GPS, Galileo, BeiDou, GLONASS, and IRNSS. The FGI-GSRx software receiver was recently released as open source for the GNSS community under the General Public License (GNU) [18]. The software receiver is intended to process raw Intermediate Frequency (IF) signals in postprocessing. The processing chain of the software receiver consists of signal acquisition, code, and carrier tracking, decoding the navigation message, pseudo-range estimation, and Position, Velocity, and Timing (PVT) computation. The software architecture is built in such a way that a new algorithm can be developed and tested at any stage in the receiver processing chain without requiring significant changes to the original codes.

The software receiver can also be utilized to develop innovative approaches for interference impact analysis, detection, and mitigation. In [19], authors implemented a Running Digital Sum (RDS)-based interference detection method in FGI-GSRx, where interference detection is conducted via a digital sum analysis of the digitized signal following the Analog-to-Digital (A/D) conversion at the IF. The RDS-based interference detection method was implemented to detect interference in GPS L1 C/A signal. Later, in [20], authors developed and tested a Narrowband Interference (NBI) detection and mitigation algorithm for GPS L1 C/A and Galileo E1 signals in FGI-GSRx. The results demonstrate that the method can identify unintentional NBI and effectively minimize the effect of NBI on GPS L1 C/A and Galileo E1 signals using the adaptive notch filtering technique. As a part of the ongoing development of FGI-GSRx, the authors of this manuscript implemented a jamming event detector by constantly monitoring the received signal strength. The implemented jamming event detection technique is computationally inexpensive, intuitive, and applicable for detection of jamming on any GNSS signal. Section 2.2 offers a detailed overview of the implemented jamming event detector. Aside from the jamming event detector implementation, other necessary modifications to FGI-GSRx were made to execute different combinations of GNSS signals in accordance with the requirements from different experimental scenarios.

### 2.2. Jamming Event Detector

**Figure 1:**
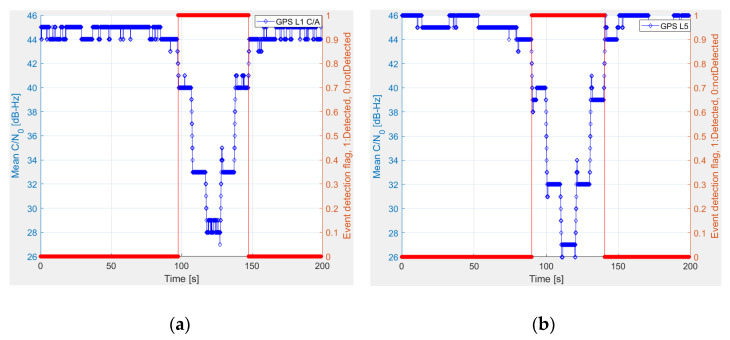
Jamming event detection for (**a**) GPS L1 C/A and (**b**) GPS L5.

The maritime environment is not usually so harsh as land in terms of satellite visibility. It is highly uncommon for all satellites to be blocked due to a lack of visibility or obstacles. In the event of poor satellite visibility or obstacle, all signals from the same satellite would be blocked. In the case of a maritime open sea environment, a sudden drop in the Carrier-to-Noise density ratio ($C/N_{0}$) of all satellites in the same frequency band is most likely some form of jamming, either intentional or unintentional. The idea here is to detect a jamming event and generate a jamming event detection flag and an alert message to the end-users based on the predefined detection threshold. The STRIKE3 project [21] proposed two standard event definitions, one of which is implemented in this manuscript. A jamming event is detected by the receiver based on the $C/N_{0}$ measurements. If the mean $C/N_{0}$ of all satellites in view drops 4 dB or more compared with the expected mean $C/N_{0}$ of all satellites in a nominal situation, then a jamming event is said to be underway. The event detector incorporates one of the most commonly used KPIs (i.e., $C/N_{0}$) in any GNSS receiver. As a result, when compared with traditional MFMC receivers, no considerable additional processing costs in detection and mitigation are expected. Figure 1 illustrates the detection of jamming on GPS L1 C/A and GPS L5 signals.

The outcome of the jamming event detector is summarized in Table 1. Jamming events are identified on the lower and upper L-bands for about 51 s on both occasions.

$C/N_{0}$ is computed at the tracking stage after each correlation period (i.e., 1 millisecond for GPS L1 C/A and 4 milliseconds for Galileo E1). A jamming event is stated to be in progress, if the following condition is met.

(1) $${Jamming}_{{Detection}_{{Flag}_{f_{signal}}}}\;=\{\begin{array}{c} 1,\;\;\;{\Delta (C/N_{0})}_{f_{signal}}\geq 4\;\mathrm{dB}\;\\ 0,\;\;\;otherwise \end{array}$$

In Equation (1),$\;∆{\left(C/N_{0}\right)}_{f_{signal}}$ is the degradation in average $C/N_{0}$ observed by the event detector for any specific GNSS signal can be expressed as follows:

(2) $$∆{\left(C/N_{0}\right)}_{f_{signal}}={\left(\bar{C/N_{0}}\right)}_{f_{{signal}_{n}}}\;-{\left(\bar{C/N_{0}}\right)}_{f_{{signal}_{j}}}$$

In Equation (2), ${\left(\bar{C/N_{0}}\right)}_{f_{{signal}_{n}}}$ is the average expected $C/N_{0}$ in nominal scenario, and ${\left(\bar{C/N_{0}}\right)}_{f_{{signal}_{j}}}$ is the measured average $C/N_{0}$ of $f_{signal}$ at the $j^{th}$ time instant. They can be expressed by the following equations:

(3) $${\left(\bar{C/N_{0}}\right)}_{f_{{signal}_{n}}}=\frac{1}{{\left(N_{sat}\right)}_{n}}\overset{{\left(N_{sat}\right)}_{n}}{\underset{k=1}{\sum }}\left(C/N_{0}{}_{n}\right){\;}_{k}$$

(4) $${\left(\bar{C/N_{0}}\right)}_{f_{{signal}_{j}}}=\frac{1}{{\left(N_{sat}\right)}_{j}}\overset{{\left(N_{sat}\right)}_{j}}{\underset{k=1}{\sum }}\left(C/N_{0}{}_{j}\right){\;}_{k}$$

In Equation (3), ${\left(C/N_{0}{}_{n}\right)}_{k}$ is the nominal $C/N_{0}$ in the absence of any deliberate jamming of the $k^{th}$ satellite, ${\left(N_{sat}\right)}_{n}$ is the total number of satellites observed by the receiver in the nominal situation. The ${\left(\bar{C/N_{0}}\right)}_{f_{{signal}_{n}}}$ is derived only once for a specific $f_{signal}$ in nominal situation.

In Equation (4), ${\left(C/N_{0}{}_{j}\right)}_{k}$ is the observed $C/N_{0}$ at the $j^{th}$ time instant of the $k^{th}$ satellite, ${\left(N_{sat}\right)}_{j}$ is the total number of satellites observed by the receiver at the $j^{th}$ time instant.

There is also a recommendation proposed in [20] to report the duration of each event (i.e., the time when interference starts and the time when it ends), which is implemented in this manuscript. For this purpose, the duration of jamming event detector is defined as follows:

(5) $$t_{j}=Jamming_{EndTime}-Jamming_{StartTime}$$

In Equation (5), $t_{j}$ is the duration of jamming in seconds. $Jamming_{StartTime}$ is defined as the time when $∆{\left(C/N_{0}\right)}_{f_{signal}}$ is either equal to or above a predefined threshold (i.e., 4 dB in this case). Similarly, $Jamming_{EndTime}$ is defined as the time when $∆{\left(C/N_{0}\right)}_{f_{signal}}$ is below the predefined threshold. However, there must always be a valid $Jamming_{StartTime}$ in order to recognize $Jamming_{EndTime}$ as a valid time. When jamming is observed on any GNSS signal, reporting a jamming incident to end-users becomes quite crucial. The end-users will be notified of a jamming incident immediately after the end of a jamming event.

### 2.3. Test Setup and Data Collection

**Figure 2:**
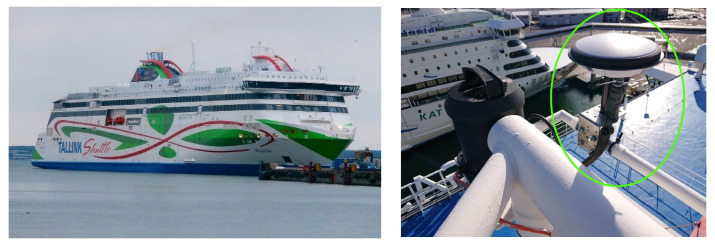
MS Megastar [23] cruise ship (**left**), GNSS antenna installed on the top mast of the vessel marked within green ellipsoid (**right**).

The equipment used for data collection includes a signal amplifier–splitter, a programmable attenuator [22], a radio front-end, a professional grade GNSS receiver, two hand-held jammers, a computer, and auxiliary components. The equipment was taken on-board the MS Megastar. A mounting position for the antenna was chosen at the top mast as shown in Figure 2 (right) in order to receive the best possible GNSS signals.

**Figure 3:**
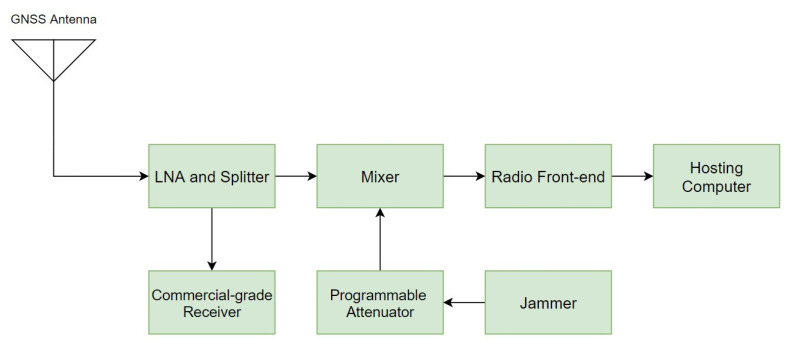
Block diagram of hardware setup used during the data collection on Megastar.

The hardware configuration for data collection is shown in Figure 3. An amplifier–splitter is used to amplify and split GNSS signals. A commercial-grade GNSS receiver is connected to one of the splitter ports, which remains unaffected by jamming and is used to compute reference trajectory. A mixer is used to combine jamming signal with clean GNSS signals. A programmable attenuator is connected between the jammers and the mixer, allowing the jamming power to be fine-tuned over time. The mixed signal is fed to the radio front-end that captures the raw GNSS signal. The radio front-end is linked via USB to a dedicated hosting computer that manages the data collection process and stores raw GNSS data.

Table 2 presents the configuration of the radio front-end used in the experiments. The GNSS signals were recorded using a TeleOrbit GTEC© dual-band RF front-end developed by Fraunhofer Institute for Integrated Circuits IIS [24]. The front-end contains two RF channels: one was configured to receive L1/E1/B1 signals at 1574.89 MHz with a bandwidth of 38 MHz, and the other one was configured to receive L5/E5a/E5b signals at 1192.50 MHz with a bandwidth of 54 MHz as mentioned in Table 2. The L1/E1/B1 signals were sampled at 40.5 MHz, while the L5/E5a/E5b signals were sampled at 81 MHz. The received samples for all signals were complex and synchronized with an internal Temperature Compensated Crystal Oscillator (TCXO) reference clock.

### 2.4. Jammer Characterization

The experiment employs two jammers to impair GNSS signals in the lower and upper L-bands. Jamming signal is injected through a cable into the front-end to keep the outside environment unaffected.

**Figure 4:**
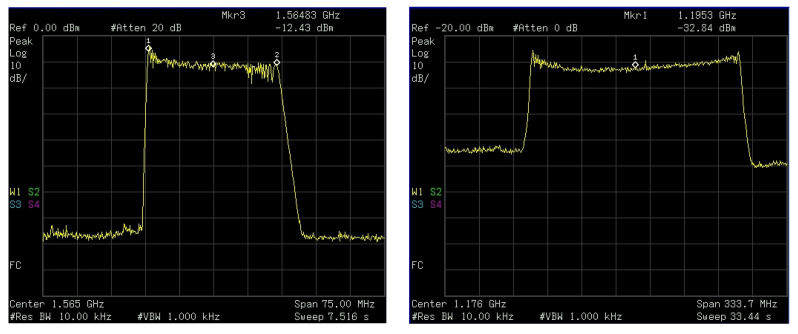
Frequency spectrum of the L1 jammer (**left**) and the L5 wideband jammer (**right**).

The upper L-band jammer (also referred as L1 jammer) transmits out a chirp signal with an approximate center frequency of 1.565 GHz and a bandwidth of 28.20 MHz. The detailed specifications of the L1 jammer can be found in [19]. The second jammer used in the jamming experiments operates in the lower L-band (also known as L5 Jammer). The spectrums of both jammers as obtained by the spectrum analyzer are shown in Figure 4. The L5 jammer is a wideband jammer with center frequency at approximately 1.1953 GHz and a bandwidth of 200 MHz, as can be seen in Table 3.

## 3. Proposed Resilient MFMC Receiver

The use of MFMC receivers in various GNSS applications is very common nowadays. MFMC improves availability and continuity while also adding redundancy to the system. However, traditional MFMC receivers are not intelligent enough to minimize positioning errors in the event of massive jamming incidents. In this manuscript, the authors propose a resilient MFMC receiver prototype that would not only benefit from traditional MFMC receivers but also improve the overall resiliency of the system by exploiting frequency diversity. The proposed resilient MFMC receiver monitors the quality of the available signal in every epoch and prepares a pass/fail criterion for each of the GNSS signals to be included for the subsequent PVT computation. The objective of the proposed MFMC prototype is to detect jamming on any GNSS signals, exclude the impacted signal(s) from subsequent PVT computation, and perhaps notify the end-user about the identified abnormalities. For example, if the system detects jamming on the GPS L1 signal, all GPS L1 pseudo-range measurements for that epoch will be omitted from the PVT computation as long as it has one GNSS signal that has the jamming-free status. In this case, the system first identifies those N number of GNSS signals with jamming-free status so that it satisfies N≥1. In the case when there are no GNSS signals with jamming-free status (i.e., in the case when N<1), the proposed resilient MFMC receiver falls back to a traditional GNSS receiver accepting all GNSS signals for PVT computation. This is to ensure that the proposed MFMC receiver makes the best use of multiple constellations and multiple frequencies in the case of simultaneous jamming on all of its received frequencies.

**Figure 5:**
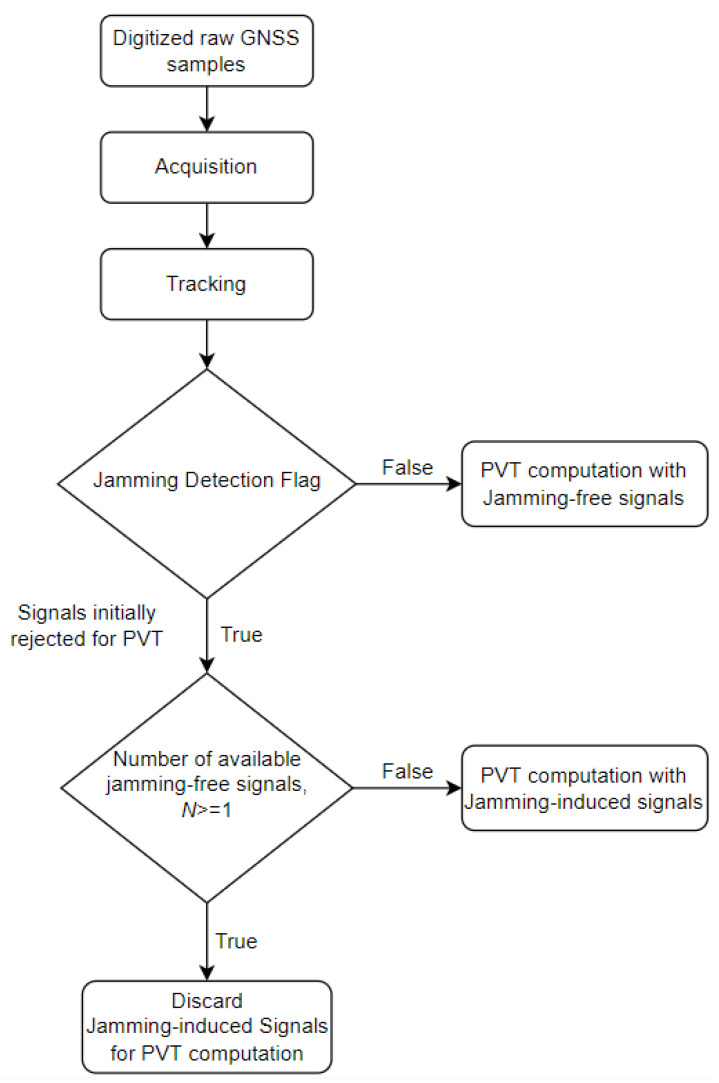
Concept illustration of a resilient MFMC receiver.

The proposed resilient MFMC receiver consists of several processing blocks, some of which are common in traditional receivers. The digitized raw GNSS samples from the front-end are processed by a signal processing chain that includes signal acquisition, code and carrier tracking, jamming detection and exclusion, and a navigation processing unit to provide a PVT solution. The working principle of the proposed resilient MFMC receiver concept is depicted in Figure 5. In the case of a single frequency jamming event either on the L1/E1/B1 or L5/E5 bands, a jamming event is detected on those impacted GNSS signals, as per Equation (1). The proposed resilient MFMC receiver excludes all jamming-induced GNSS signals from PVT computation for the entire duration of the jamming event since it has the possibility to switch to other jamming-free GNSS signals (i.e., in both of the mentioned single-frequency jamming cases, there always exists N number of jamming-free GNSS signals that satisfies N≥1). In the case of simultaneous multi-frequency jamming on all of its received GNSS signals, there is no available jamming-free signal for PVT computation (in the case when N<1). In this extreme multi-frequency jamming scenario, the proposed MFMC receiver then accepts all jamming-induced signals to be used in the PVT computation and notifies the end-user about the detected multi-signal jamming incident. As an efficient countermeasure, the receiver might seek an interference mitigation solution either internally within the receiver via receiver signal processing algorithms such as adaptive notch filters or externally by integrating an Inertial Measurement Unit (IMU) with sensor fusion [25,26,27]. However, in this manuscript, the authors emphasized the potential of a resilient MFMC receiver that outperforms a traditional GNSS receiver by intelligently switching to jamming-free signals in the event of single-frequency or single-constellation jamming.

## 4. Test Scenarios

Different scenarios with and without jamming are explored to investigate GNSS vulnerabilities in the maritime environment. When the signals are jammed, different front-ends react differently depending on the Jamming-to-Signal power ratio ((J/S). The Automatic Gain Control (AGC) of the front-end is the first line of defense against the jamming or interference [28,29]. Some front-ends may have a light interference mitigation feature built in. Since jamming signal raises the noise level in the system, it has a direct impact on the $C/N_{0}$ of the received GNSS signals.

The impact of jamming on all tracked satellites in the impacted frequency band is nearly identical for a given constellation. In the following experiments, the rapid $C/N_{0}$ degradation is observed in the affected frequency bands due to deliberate jamming, where very little or no $C/N_{0}$ variation is observed in the non-affected bands. Four different scenarios are considered to analyze the performance of a resilient MFMC receiver in a maritime environment:

- Scenario I: Nominal maritime navigation without the presence of jamming.
- Scenario II: Maritime navigation under the influence of jamming on GNSS L1/E1/B1 signal.
- Scenario III: Maritime navigation under the influence of jamming on GNSS L5/E5a/E5b signal.
- Scenario IV: Maritime Navigation Under the Influence of Jamming on Lower and Upper L-bands.

### 4.1. Scenario I: Nominal Maritime Navigation without the Presence of Jamming

**Figure 6:**
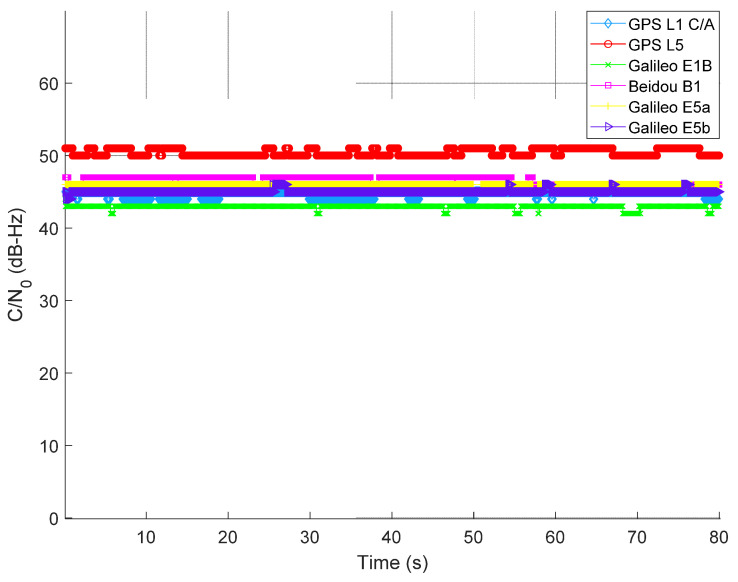
Average $C/N_{0}$ of tracked GNSS signals without the presence of intentional jamming.

In all scenarios, GNSS data were collected in an open sea environment between the Helsinki–Tallinn ferry route. The nominal situation refers to the dataset that is free of any intentional jamming. Average $C/N_{0}$ is computed for all the tracked satellites per GNSS signal. Figure 6 illustrates the computed average $C/N_{0}$ of all tracked GNSS signals, ranging between 43 and 50 dB-Hz depending on the GNSS signals.

### 4.2. Scenario II: Maritime Navigation under the Influence of Jamming on GNSS L1/E1/B1 Signal

**Figure 7:**
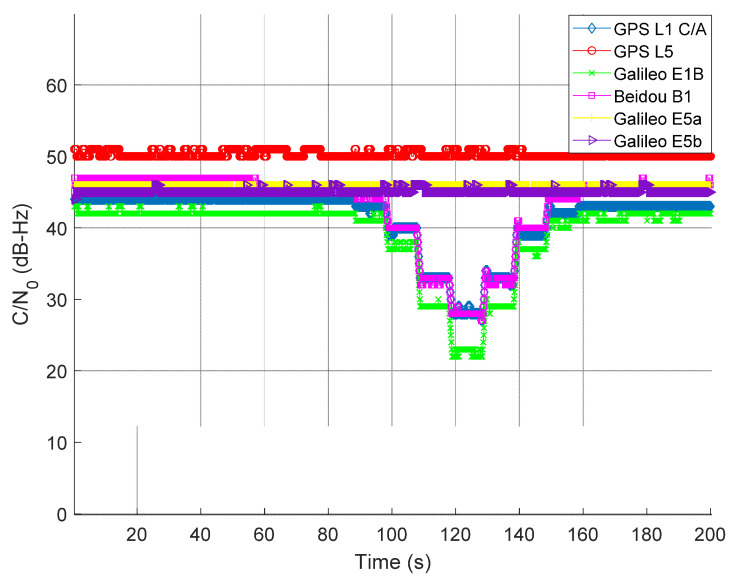
Average $C/N_{0}$ of tracked GNSS signals with the presence of jamming on L1/E1/B1 signals.

The L1 jammer was used to impair the L1/E1/B1 signals in this experiment. The jamming power was adjusted using a programmable attenuator. Intentional jamming started at around 90th second from the beginning of the experiment. The jamming power was increased with a step of 10 dB after each 10 s interval until around 120 s. At 130 s, the process was reversed (i.e., the jamming power was decreased by 10 dB after each 10 s interval until around 160 s). For the jamming on L5/E5a/E5b in Scenario III, the data collection and jamming process was identical. As seen from Figure 7, the average $C/N_{0}$ of L1/E1/B1 signal experienced a drop each time J/S power increased. The degradation of $C/N_{0}$ is quite significant at 120 s where the J/S power is at its maximum. In this experimental scenario, the L5/E5a/E5b signals remain unaffected, as seen in Figure 7.

### 4.3. Scenario III: Maritime Navigation under the Influence of Jamming on GNSS L5/E5a/E5b Signal

**Figure 8:**
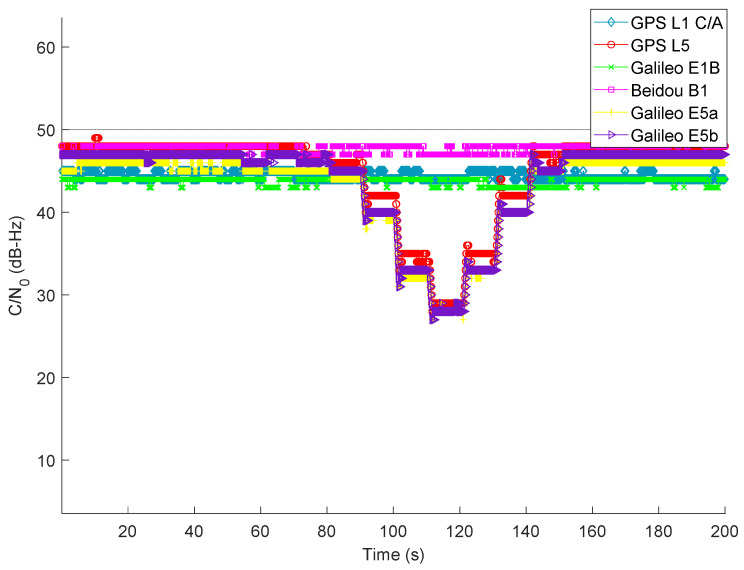
Average $C/N_{0}$ of tracked GNSS signals during the presence of jamming on L5/E5a/E5b signals.

The wideband L5 jammer was used in this scenario to impair the L5/E5a/E5b signals. Once the jamming was applied on L5/E5a/E5b signals, other signals were unaffected, as expected. As seen in Figure 8, with the variation in J/S power, the L5/E5a/E5b signal exhibits a somewhat similar average $C/N_{0}$ disparity pattern to that in Scenario II.

### 4.4. Scenario IV: Maritime Navigation under the Influence of Jamming on Lower and Upper L-Bands

**Figure 9:**
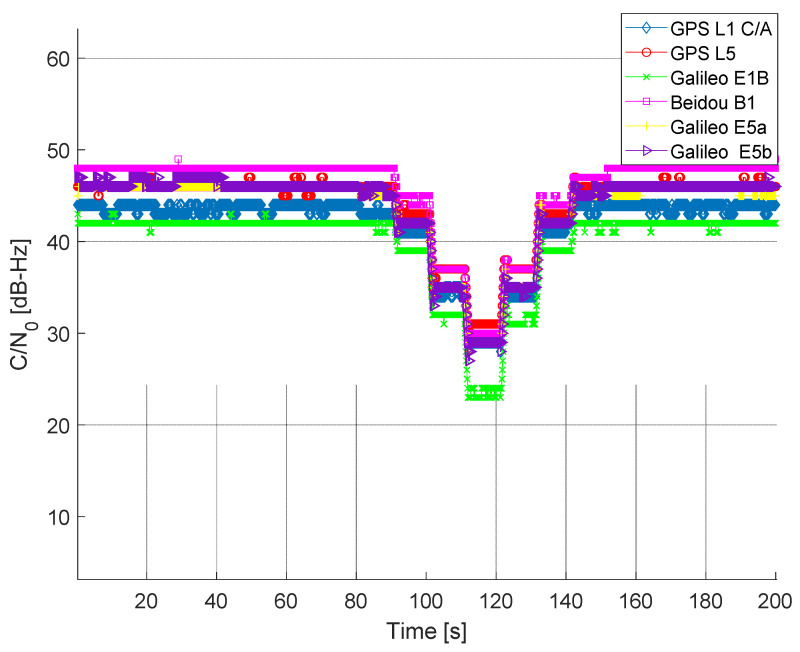
Average $C/N_{0}$ of tracked GNSS signals during the presence of jamming in lower and upper L-band.

In this experiment, both L1 and L5 wideband jammers are applied to interfere with the lower and upper L-bands. A separate mixer is utilized to integrate the jamming signals from both jammers, which are then mixed with the clean GNSS signal. The jamming profile stays unchanged from earlier experiments (i.e., Scenarios II and III). A full ramp jamming profile is used, starting at the 90th second and ending at the 150th second, with a 10 dB step. As illustrated in Figure 9, all signals begin to experience degraded $C/N_{0}$ at the 90th second and keep following the whole ramp pattern until the 150th second. For all signals around the 120th second, the $C/N_{0}$ degradation is very significant. All signals are recovered almost instantly once the jamming signals are removed.

## 5. Results

This section starts with an overview of the reference trajectory generation procedure for the test campaign. Afterwards, a comparative performance analysis is presented for the four scenarios mentioned in Section 4. The key findings of the analysis are highlighted with figures and tables.

### 5.1. Reference Trajectory Generation

The reference trajectory was generated using a professional-grade NovAtel ProPak6™ GNSS receiver. The position estimates of the receiver were enhanced with RTK corrections computed using raw observation data from GNSS ground reference stations in Finland (belonging to the Finnish Permanent GNSS Network, FinnRef) and Estonia (belonging to the Estonian permanent GNSS network, ESTREF). The GNSS+RTK combination was performed in post mission utilizing a software tool Inertial Explorer^®^ (IE) from NovAtel Inc., Calgary, AB, Canada. Four stations (with station identification MET3, SUR4, MUS2, and KUSA) cover the entire sea trajectory between Helsinki and Tallinn.

### 5.2. Positioning Performance Analysis

Raw IF samples of GNSS signals were collected on 26 August 2021, between 8:00 to 10:00 UTC on the Helsinki–Tallinn ferry route in the Gulf of Finland. The gathered datasets were then analyzed in post mission using the FGI-GSRx MFMC software receiver under various signal combinations for all four scenarios, as described in Section 4. Position solution was computed with a 15-degree elevation mask and 20 dB-Hz $C/N_{0}$ threshold. This means that the satellite elevation has to be above 15 degrees and the $C/N_{0}$ has to be above 20 dB-Hz in order for the satellite to be included in the position computation. Besides, position solution was computed at 1 Hz rate using a Least Squares (LS) algorithm. Positioning accuracy of horizontal, vertical, and 3-Dimentional Root-Mean-Square (RMS) error is presented below in Table 4, Table 5, Table 6 and Table 7 for Scenarios I, II, III, and IV, respectively.

Table 4 shows positioning performance of GPS, Galileo, and BeiDou for each signal component, a combined single-frequency multi-constellation, and MFMC solution. As in Table 4, Galileo E5a has the best 3D positioning performance among all the analyzed signals. Galileo E5b offers almost similar positioning performance as of E5a with a slightly higher PDOP (Position Dilution of Precision describes the inaccuracy caused by the relative position of the navigation satellite and the user) than Galileo E5a.

Since there is no intentional jamming applied in this case, both MFMC (i.e., combination of all six signals) and the proposed resilient MFMC offers the best horizontal accuracy with an excellent PDOP (<1) among all the considered signals and their combinations. As expected, Galileo E5a, Galileo E5b, and GPS L5 individually offer better horizontal positioning accuracy over all other signals even with relatively high PDOP values. It is observed that the positioning performance of GPS L5 is slightly worse than expected, which can be attributed to a fewer number of satellites in the final position solution.

Table 5 presents the positioning performance of GPS, Galileo, and BeiDou for Scenario II. The test window (i.e., the duration of interest out of the whole data length) of the dataset is estimated based on the output of the jamming event detector. The Positioning solution is computed for the data duration when the jamming signal is detected to be present. The jamming start time and end time estimated by the detector for Scenario II are at the 97th and 148th second, respectively, resulting in a jamming period of 51 s, as shown in Table 1. The test window of unaffected signals is also kept the same in order to offer a fair comparison.

As seen in Table 5, GPS L1 C/A is the most severely affected, followed by BeiDou B1 and Galileo E1. Furthermore, multi-constellation (in this case, L1/E1/B1) performs better than each of the individual signals at the time of jamming, demonstrating the importance of having multiple constellations for minimizing the jamming impact on the positioning accuracy. Signals in L5, E5a, and E5b bands, on the other hand, offer expected positioning performance since the L5/E5 frequency band is completely unaffected by jamming. Positioning accuracy of Galileo E5a and E5b is steady with a 3D-RMS of roughly 2 m. Better horizontal accuracy with the combination of GPS L5, Galileo E5a, and Galileo E5b can also be observed from Table 5.

Table 6 presents the positioning performance of Scenario III. Similarly, the test window for affected and unaffected signals is kept the same. Since deliberate jamming is carried out on the L5/E5a/E5b bands, it is expected that the jamming influence would be seen mostly on the L5/E5a/E5b bands.

As shown in Table 6, despite being jammed, the positioning performance of GPS L5, Galileo E5a, and Galileo E5b signals is relatively stable and slightly degraded compared with the jamming-free situation in Scenario I. Larger code length, higher chipping rate, along with higher receiver bandwidth increase the robustness of L5/E5a/E5b signals against wideband jamming, resulting in less performance degradation during jamming when compared with the L1/E1/B1 signals in Scenario II.

Table 7 shows the positioning performance of several GNSS signals where both jammers are effectively jamming both the upper and lower L-bands simultaneously. Since jamming is induced on both the lower and upper L-bands, a higher positioning error is expected compared with the nominal situation. As can be observed in Table 7, all the signals perform worse than the nominal situation. GPS L1 C/A is highly impacted during the jamming event compared with other signals. On the other hand, the positioning performance of Galileo E5a and E5b remain consistent with Scenario III.

Based on the results in Table 7 and other analyses presented in this manuscript, it can be stated that higher modulation order and longer codes coupled with higher reception bandwidth allow the L5/E5 band to operate reliably better than L1/E1/B1 band in the event of moderate jamming. However, a fair comparison of jamming impact analysis on each GNSS signal can be made only when the jamming source is identical to both the signals, which is not the case for this particular experiment.

**Figure 10:**
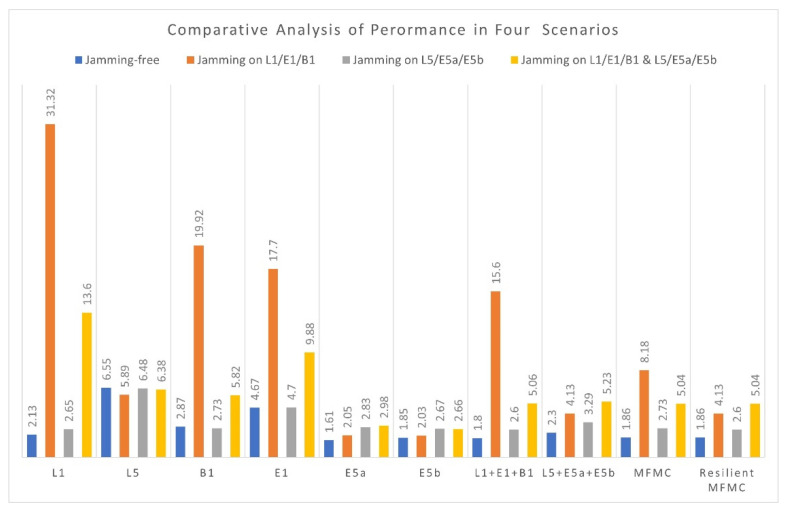
3D-RMS comparison of different GNSS signals under four scenarios.

Figure 10 shows a summary of the 3D-positioning performance of different GNSS signals for the mentioned four scenarios. As can be seen from Figure 10, overall, jamming has a lower impact on GPS L5, Galileo E5a, and Galileo E5b than it does on GPS L1 C/A, Galileo E1, and BeiDou B1. Furthermore, in the nominal situation, signals in the L5/E5 band provide better 3D-positioning performance than signals in the L1/E1/B1 band.

Figure 10 also demonstrates that single-frequency multi-constellation diversity improves receiver resiliency by leveraging diverse characteristics of contemporary multi-constellation signals. Finally, when it comes to mitigation of jamming impact and enhancing the robustness of the GNSS receivers, a resilient MFMC solution with a jamming event detector is always viable.

**Figure 11:**
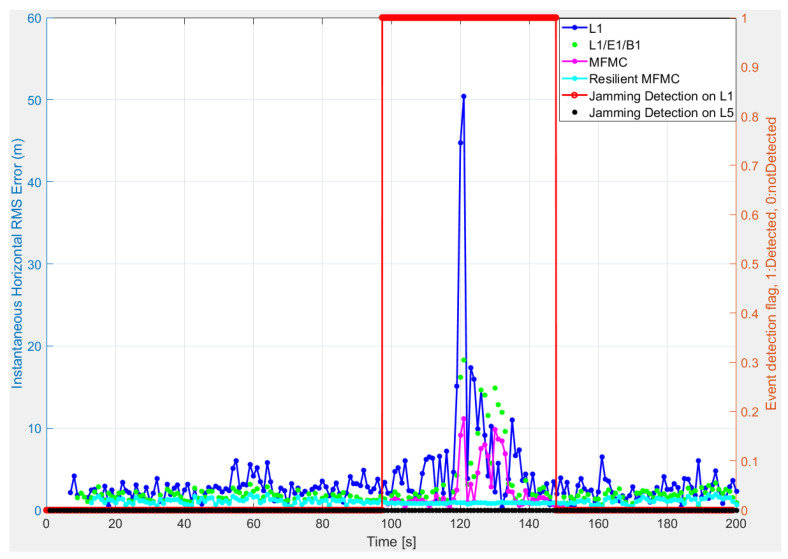
Instantaneous horizontal position error of different signal combinations along with jamming event detection for Scenario II.

As an example of how the resilient MFMC receiver performed in a maritime environment for the whole test duration (i.e., ~200 s), Figure 11 offers a comparison of instantaneous horizontal positioning accuracy for Scenario II. FGI-GSRx can generate a PVT solution with reference to the beginning of the first subframe of each signal, which caused different initial delays for different signals in obtaining the positioning solution. These initial delays of not having PVT solution can be seen in Figure 11. It can be observed that the proposed resilient MFMC receiver first utilized all the available signals and all constellations until jamming was detected on L1/E1/B1 signals; after this, it switched from traditional MFMC to single-frequency, multi-constellation solution utilizing jamming-free L5/E5a/E5b signals for the duration of jamming on L1/E1/B1; then, later it again switched back to normal MFMC-based position solution, once there was no more jamming detected on L1/E1/B1 bands. The proposed resilient MFMC receiver provided an uninterrupted positioning solution in the case of a single-frequency jamming incident. A similar finding was also observed for Scenario III.

## 6. Conclusions

The authors implemented a jamming event detector-based resilient MFMC software receiver that exploits frequency diversity to combat single-frequency jamming. It was demonstrated that the proposed resilient MFMC receiver offers better positioning accuracy than any single-constellation receiver in the case of single-frequency jamming. The resilient receiver can combat jamming in either the L1/E1/B1 or the L5/E5a/E5b frequency bands by properly detecting jamming on the affected signals and then excluding those signals while computing the PVT solution. Finally, the authors demonstrated the positioning performance of MFMC receiver in an operational maritime environment along with a detailed performance assessment of GPS-L5-only, Galileo-E5a-only, and Galileo-E5b-only signals that is assumed to be very novel for a research receiver. It was observed that the Galileo E5a or Galileo E5b position solutions outperform the L1/E1/B1-only position solution in nominal situation due to the improved signal characteristics in the L5/E5 frequency band. In this example dataset, the GPS L5 signal had relatively poor satellite visibility that contributed to the ultimate position accuracy. However, it is expected that GPS L5 will offer similar positioning performance to that of Galileo E5a/E5b signals when it reaches its full constellation in the future.

The navigation results demonstrate the true potential of utilizing frequency diversity to combat jamming when it occurred in one of the GNSS frequencies. In general, following are the core contributions of this work:

- Implementation of a jamming event detector to identify jamming on any GNSS signals.
- Implementation and validation of a novel resilient MFMC receiver to combat jamming on any single-frequency band.
- The first navigation results of single-frequency GPS L5, Galileo E5a, and Galileo E5b signals in a maritime operational environment utilizing an in-house research receiver.

Future work includes impact analysis of jamming on Galileo E5 full-band AltBOC signal. To the authors’ knowledge, impact analysis of jamming on Galileo E5 full-band AltBOC signal has not yet been thoroughly studied in the literature.

## Figures and Tables

**Table 1:** Jamming event detection on different GNSS signals.

L1/E1/B1				L5/E5a/E5b		
Signals	Event Start (s)	Event End (s)	Duration (s)	Event Start (s)	Event End (s)	Duration (s)
L1	97	148	51	-	-	-
E1	97	148	51	-	-	-
B1	97	148	51	-	-	-
L5	-	-	-	90	141	51
E5a	-	-	-	90	141	51
E5b	-	-	-	90	141	51

**Table 2:** Front-end configuration for TeleOrbit GTEC© dual-band GNSS signal receiver.

Parameters	Frequency Bands (L1/E1/B1)	Frequency Bands (L5/E5a, E5b)
Center frequency (MHz)	1574.890625	1192.50
Sampling rate (MHz)	40.5	81
Data type	Complex	Complex
Sample bit width	8 bit + 8 bit (I + Q)	8 bit + 8 bit (I + Q)
Bandwidth (MHz)	38	54

**Table 3:** Jamming signal parameters.

Parameters	L1 Jammer	L5 Wideband Jammer
Center frequency (MHz)	1564.74	1195.30
Bandwidth (MHz)	28.20	200
Impacted GNSS signals	L1/E1/B1	Lower L-band

**Table 4:** Positioning accuracy of Scenario I with different signal combinations.

Scenario I (Nominal Situation)					
GNSS Signals	Horizontal RMS (m)	Vertical RMS (m)	3D-RMS (m)	PDOP	$N_{sat}$
L1	1.69	1.30	2.13	2.41	6
L5	1.87	6.27	6.55	3.89	5
B1	2.71	0.95	2.87	2.35	7
E1	2.71	3.80	4.67	2.93	6
E5a	1.44	0.72	1.61	2.37	7
E5b	1.57	0.97	1.85	2.93	6
L1 + E1 + B1	0.89	1.56	1.80	1.29	19
L5 + E5a + E5b	0.84	2.14	2.30	1.37	18
MFMC (L1 + E1 + B1 + L5 + E5a + E5b)	0.53	1.78	1.86	0.93	37
Resilient MFMC	0.53	1.78	1.86	0.93	37

**Table 5:** Positioning accuracy of Scenario II with different signal combinations.

Scenario II (Jamming on L1/E1/B1 Band)					
GNSS Signals	Horizontal RMS (m)	Vertical RMS (m)	3D-RMS (m)	PDOP	$N_{sat}$
L1	13.27	28.36	31.32	2.12	7
L5	4.95	3.18	5.89	4.80	5
B1	12.74	15.31	19.92	2.79	7
E1	11.43	13.51	17.70	2.63	7
E5a	1.93	0.67	2.05	2.63	7
E5b	1.94	0.61	2.03	2.63	7
L1+ E1 + B1	6.44	14.20	15.60	1.29	21
L5 + E5a + E5b	0.88	4.03	4.13	1.53	19
MFMC (L1 + E1 + B1 + L5 + E5a + E5b)	3.29	7.49	8.18	0.97	40
Resilient MFMC	0.88	4.03	4.13	1.53	19

**Table 6:** Positioning accuracy of Scenario III with different signal combinations.

Scenario III (Jamming on L5/E5a/E5b Band)					
GNSS Signals	Horizontal RMS (m)	Vertical RMS (m)	3D-RMS (m)	PDOP	$N_{sat}$
L1	1.17	2.37	2.65	2.41	6
L5	2.04	6.15	6.48	3.90	5
B1	2.47	1.18	2.73	2.35	7
E1	3.30	3.35	4.70	2.91	6
E5a	2.03	1.97	2.83	2.38	7
E5b	1.81	1.97	2.67	2.91	6
L1 + E1 + B1	0.78	2.47	2.60	1.29	19
L5 + E5a + E5b	1.05	3.12	3.29	1.36	18
MFMC (L1 + E1 + B1 + L5 + E5a + E5b)	0.782	2.62	2.73	0.92	37
Resilient MFMC	0.78	2.47	2.60	1.29	19

**Table 7:** Positioning accuracy of Scenario IV with different signal combinations.

Scenario IV (Jamming on Both L1/E1/B1 and L5/E5a/E5b Bands)					
GNSS Signals	Horizontal RMS (m)	Vertical RMS (m)	3D-RMS (m)	PDOP	$N_{sat}$
L1	6.29	12.05	13.60	1.69	8
L5	2.91	5.68	6.38	4.22	5
B1	2.63	5.19	5.82	3.40	6
E1	5.87	7.94	9.88	2.00	8
E5a	1.73	2.42	2.98	2.00	8
E5b	1.78	1.98	2.66	2.12	7
L1 + E1 + B1	3.14	3.97	5.06	1.13	22
L5 + E5a + E5b	0.86	5.16	5.23	1.25	20
MFMC (L1 + E1 + B1 + L5 + E5a + E5b)	2.19	4.53	5.04	0.84	42
Resilient MFMC	2.19	4.53	5.04	0.84	42

## Data Availability

Not applicable.
